# Analytical Analysis of Heat Transfer and Entropy Generation in a Tube Filled with Double-Layer Porous Media

**DOI:** 10.3390/e22111214

**Published:** 2020-10-26

**Authors:** Kun Yang, Wei Huang, Xin Li, Jiabing Wang

**Affiliations:** School of Energy and Power Engineering, Huazhong University of Science and Technology, Wuhan 430074, China; yangk@hust.edu.cn (K.Y.); 13035115213@163.com (W.H.); m201971199@hust.edu.cn (X.L.)

**Keywords:** porous media, double-layer, local thermal non-equilibrium, analytical solution

## Abstract

The heat transfer and entropy generation in a tube filled with double-layer porous media are analytically investigated. The wall of the tube is subjected to a constant heat flux. The Darcy-Brinkman model is utilized to describe the fluid flow, and the local thermal non-equilibrium model is employed to establish the energy equations. The solutions of the temperature and velocity distributions are analytically derived and validated in limiting case. The analytical solutions of the local and total entropy generation, as well as the Nusselt number, are further derived to analyze the performance of heat transfer and irreversibility of the tube. The influences of the Darcy number, the Biot number, the dimensionless interfacial radius, and the thermal conductivity ratio, on flow and heat transfer are discussed. The results indicate, for the first time, that the Nusselt number for the tube filled with double-layer porous media can be larger than that for the tube filled with single layer porous medium, while the total entropy generation rate for the tube filled with double-layer porous media can be less than that for the tube filled with single layer porous medium. And the dimensionless interfacial radius corresponding to the maximum value of the Nusselt number is different from that corresponding to the minimum value of the total entropy generation rate.

## 1. Introduction

Porous media has considerable advantages of large specific surface area and complex pore structure, which makes the porous media have excellent heat transfer performance and extensive range of industrial applications, such as sewage treatment, electronic device cooling, fuel cells, solar collectors, compact heat exchanger, and heat transfer enhancement.

Heat transfer and transport phenomenon in porous media has gained increasing attention. Kim et al. [1] derived the analytical solution for temperature field in the microchannel heat sink. Jing et al. [2,3] theoretically and numerically studied the flow and heat transfer in tree-like branching microchannel. Pavel and Mohamad [4] conducted experimental work to investigate the influence of inserting porous media on heat transfer rate within a tube. Lu et al. [5] theoretically study the effects of porosity and pore size on heat transfer in a pipe filled with high porosity porous media. Yang and Vafai [6,7] analyzed the phenomenon of heat flux bifurcation in a channel filled with porous media. Xu et al. [8] studied the forced convection in a porous parallel-plate channel using a modified fin analysis method. Dehghan et al. [9] investigated the developed region in a porous tube using perturbation techniques to study the influence of local thermal non-equilibrium (LTNE) condition. Zhang et al. [10] semi-analytically studied the flow and heat transfer in a porous tube with the inertia term, thermal dispersion and axial conduction ignored, and numerically analyzed the influences of the neglected terms. Dehghan et al. [11] studied the influences of the thermal conductivity varying with temperature on heat transfer in a porous parallel-plate channel using perturbation methods. Yang et al. [12] analytically analyzed two approaches to describe the adiabatic boundary condition and the heat flux bifurcation phenomenon for a porous parallel-plate channel. Torabi et al. [13,14,15] and Yang et al. [16] investigated the heat transfer and irreversibility in a parallel-plate channel partially filled with single-layer porous media and N-layer porous medium, respectively. Yang et al. [17] and Mahmoudi et al. [18] also investigated the heat transfer in a channel partially filled with porous media. Mohamed et al. [19] explored the influences of essential parameters on temperature profiles in a circular cylinder filled with porous media. Buonomo et al. [20] investigated the fully developed forced convection in parallel plates porous micro-channels. Based on the above studies, it can be concluded that porous media insert can effectively enhance heat transfer.

To analyze the effect of the heterogeneity of porous media on heat transfer, the channel filled with layered porous media was investigated. Nield and Kuznetsov [21] studied the influences of transverse variation of permeability and thermal conductivity on heat transfer in a channel filled with double-layer porous media based on Darcy equation and local thermal equilibrium (LTE) model. Nield and Kuznetsov extended their work to the case of asymmetric property variation and heating [22]. Nield et al. [23] and Sundaravadivelu et al. [24] investigated the influence of the viscosity varying with temperature on heat transfer in a channel filled with layered porous media. Nield and Kuznetsov [25] studied the interaction between the transverse heterogeneity of porous media and thermal development in a channel filled with layered porous media. Based on the above theoretical studies, the optimization of heat transfer performance in a channel filled with layered porous media was studied [26,27,28,29].

Most above-mentioned works for the channel filled with layered porous media were studied using Darcy equation and LTE model. However, the Darcy equation and LTE model neglect the viscous effect of impermeable wall and the heat exchange between two phases in porous media, respectively. To solve the problem, the Darcy-Brinkman equation and the local thermal non-equilibrium (LTNE) model can be used. Misra et al. [30] numerically studied the temperature distribution and entropy generation in a microfluidic tube using Darcy-Forchheimer-Brinkman equation. Nield and Kuznetsov analytically analyzed the effects of the transverse heterogeneity on heat transfer in a channel filled with layered porous media using Darcy-Brinkman equation [31] and LTNE model [32]. However, to the best of authors’ knowledge, there are no related analytical works which utilized Darcy-Brinkman equation and LTNE model simultaneously published in the literature. Entropy generation is the key thermodynamic parameter to measure the irreversibility of the flow and heat transfer process. The smaller entropy generation within the system, the less energy is destructed [13,14,15]. However, there are few studies on the entropy generation in a compositive porous channel.

The main objective of this paper was to analytically investigate the heat transfer and entropy generation in a tube filled with double-layer porous media for the engineering applications including heat transfer enhancement and electronic device cooling. The constant heat flux boundary condition was applied on the wall of the tube. The Darcy-Brinkman equation and the LTNE model were adopted to establish momentum and energy equations. The analytical solutions of the temperature and velocity distributions were derived, and the analytical solutions of the Nusselt number, the local, and total entropy generation rate were further obtained. The influences of pertinent parameters were discussed. It was found for the first time that the performance of heat transfer for the tube filled with double-layer porous media can be better than that for the tube filled with single layer porous medium.

## 2. Problem Descriptions

### 2.1. Physical Problem

**Figure 1:**
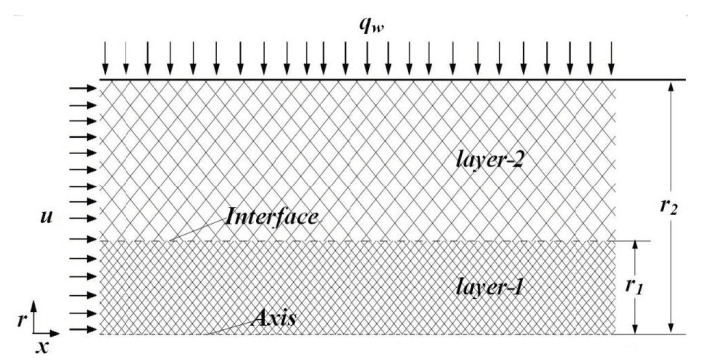
Geometry diagram of the tube filled with double-layer porous media and the corresponding coordinate system.

The schematic diagram of the tube studied in this study is shown in Figure 1. The radius of *layer-1* is $r_{1}$, and the radius of tube is $r_{2}$. The wall of the tube is subjected to a constant heat flux, $q_{w}$. Due to the symmetry, only half of the tube is considered in this work. The following assumptions are invoked in analyzing this problem:

Each porous layer is homogenous and isotropic.
The flow and heat transfer are steady and fully developed.
The pertinent physical properties are constant.
The gravity is neglected.
The no-slip condition is used for the velocity boundary condition.
The internal heat source in the energy equation is not considered.

### 2.2. Governing Equations

Based on the above assumptions, the governing equations of the fluid flow and heat transfer are obtained.

The momentum equations are described by the Darcy-Brinkman model [33],

(1) $$\mathrm{layer-1}:\;-\frac{dp}{dx}+\frac{\mu_{f}}{\varepsilon_{1}}\frac{1}{r}\frac{\partial }{\partial r}(r\frac{\partial u}{\partial r})-\frac{\mu_{f}}{K_{1}}u=0\;\left(0<r<r_{1}\right),$$

(2) $$\mathrm{layer-2}:\;-\frac{dp}{dx}+\frac{\mu_{f}}{\varepsilon_{2}}\frac{1}{r}\frac{\partial }{\partial r}(r\frac{\partial u}{\partial r})-\frac{\mu_{f}}{K_{2}}u=0\;\left(r_{1}<r<r_{2}\right).$$

Energy equations of fluid phase are as follows [33]:

(3) $$\mathrm{layer-1}:\;k_{fe1}(\frac{\partial^{2}T_{f}}{\partial r^{2}}+\frac{1}{r}\frac{\partial T_{f}}{\partial r})+h_{1}a_{1}(T_{s}-T_{f})=\rho_{f}c_{f}u\frac{\partial T_{f}}{\partial x}\;(0<r<r_{1}),$$

(4) $$\mathrm{layer-2}:\;k_{fe2}(\frac{\partial^{2}T_{f}}{\partial r^{2}}+\frac{1}{r}\frac{\partial T_{f}}{\partial r})+h_{2}a_{2}(T_{s}-T_{f})=\rho_{f}c_{f}u\frac{\partial T_{f}}{\partial x}\;(r_{1}<r<r_{2}).$$

Energy equations of solid phase are as follows [33]:

(5) $$\mathrm{layer-1}:\;k_{se1}(\frac{\partial^{2}T_{s}}{\partial r^{2}}+\frac{1}{r}\frac{\partial T_{s}}{\partial r})-h_{1}a_{1}(T_{s}-T_{f})=0\;(0\;<\;r<r_{1}),$$

(6) $$\mathrm{layer-2}:\;k_{se2}(\frac{\partial^{2}T_{s}}{\partial r^{2}}+\frac{1}{r}\frac{\partial T_{s}}{\partial r})-h_{2}a_{2}(T_{s}-T_{f})=0\;(r_{1}\;<\;r<r_{2}).$$

For thermally developed flow, the following equation is obtained based on energy balance within the tube.

(7) $$\frac{\partial T_{f}}{\partial x}=\frac{dT_{fb}}{dx}=\frac{2q_{w}}{\rho_{f}c_{f}u_{m}r_{2}}.$$

By substituting Equation (7) into Equations (3) and (4), Equations (8) and (9) are obtained.

(8) $$\mathrm{layer-1}:\;k_{fe1}(\frac{\partial^{2}T_{f}}{\partial r^{2}}+\frac{1}{r}\frac{\partial T_{f}}{\partial r})+h_{1}a_{1}(T_{s}-T_{f})=\frac{2q_{w}}{r_{2}}\frac{u}{u_{m}}\;(0<r<r_{1}),$$

(9) $$\mathrm{layer-2}:\;k_{fe2}(\frac{\partial^{2}T_{f}}{\partial r^{2}}+\frac{1}{r}\frac{\partial T_{f}}{\partial r})+h_{2}a_{2}(T_{s}-T_{f})=\frac{2q_{w}}{r_{2}}\frac{u}{u_{m}}(r_{1}<r<r_{2}),$$

where $u_{m}$ is written as

(10) $$u_{m}=\frac{1}{\pi r_{2}^{2}}\left(\int_{0}^{r_{1}}u_{1}\cdot 2\pi r\cdot dr+\int_{r_{1}}^{r_{2}}u_{2}\cdot 2\pi r\cdot dr\right).$$

### 2.3. Boundary Conditions

The boundary condition at the wall for momentum equations and energy equations are represented as follows [5]:

(11) $$u=0\;(r=r_{2}),$$

(12) $$q_{w}=-k_{fe2}\frac{\partial T_{f}}{\partial r}-k_{se2}\frac{\partial T_{s}}{\partial r}\;T_{f}=T_{s}=T_{w}\;(r=r_{2}).$$

Due to the symmetry configuration, the following boundary condition can be used at the axis of the tube.

(13) $$\frac{\partial u}{\partial r}=0\frac{\partial T_{s}}{\partial r}=\frac{\partial T_{f}}{\partial r}=0\;(r=0).$$

At the interface between two layers porous media, the continuities of the velocity, shear stress, temperature of solid and fluid, heat flux of solid, and fluid are applied [16,34,35,36].

(14) $$u_{1}=u_{2}\;\frac{1}{\varepsilon_{1}}\frac{\partial u}{\partial r}=\frac{1}{\varepsilon_{2}}\frac{\partial u}{\partial r}\;T_{f1}=T_{f2}\;k_{fe1}\frac{\partial T_{f}}{\partial r}=k_{fe2}\frac{\partial T_{f}}{\partial r}\;T_{s1}=T_{s2}\;k_{se1}\frac{\partial T_{s}}{\partial r}=k_{se2}\frac{\partial T_{s}}{\partial r}\;(r=r_{1})$$

### 2.4. Normalization

The following dimensionless parameters are introduced to normalize the governing equations and boundary conditions:

(15) $$\begin{array}{l} U=\frac{u}{u_{m}}\;R=\frac{r}{r_{2}}\;R_{1}=\frac{r_{1}}{r_{2}}\;P=\frac{dp}{dx}\frac{r_{2}^{2}}{\mu_{f}u_{m}}\;Da_{1}=\frac{K_{1}}{r_{2}^{2}}\;Da_{2}=\frac{K_{2}}{r_{2}^{2}}\;\theta_{s}=\frac{T_{s}-T_{w}}{q_{w}r_{2}/k_{se1}}\\ \theta_{f}=\frac{T_{f}-T_{w}}{q_{w}r_{2}/k_{se1}}\;Bi_{1}=\frac{h_{1}a_{1}r_{2}^{2}}{k_{se1}}\;Bi_{2}=\frac{h_{2}a_{2}r_{2}^{2}}{k_{se1}}\;k_{0}=\;\frac{k_{f}}{k_{se1}}\;k_{1}=\frac{k_{fe1}}{k_{se1}}\;k_{2}=\frac{k_{fe2}}{k_{se1}}\;\\ k_{s1}=1\;k_{s2}=\frac{k_{se2}}{k_{se1}}\;s_{1}=\sqrt{\frac{\varepsilon_{1}}{Da_{1}}}\;s_{2}=\sqrt{\frac{\varepsilon_{2}}{Da_{2}}}\;t_{1}=\sqrt{\frac{k_{1}+k_{s1}}{k_{1}k_{s1}}Bi_{1}}\;t_{2}=\sqrt{\frac{k_{2}+k_{s2}}{k_{2}k_{s2}}Bi_{2}}\\ F=\frac{T_{w}k_{se1}}{q_{w}r_{2}}\;Pe=\frac{c_{f}\rho_{f}r_{2}u_{m}}{2k_{se1}}\;Br=\frac{\mu_{f}u_{m}^{2}}{q_{w}r_{2}} \end{array}$$

The momentum Equations (1) and (2) are rewritten as

(16) $$\mathrm{layer-1}:\;\frac{\partial^{2}U}{\partial R^{2}}+\frac{1}{R}\frac{\partial U}{\partial R}\;=\varepsilon_{1}(P+\frac{U}{Da_{1}})\;(0<R<R_{1}),$$

(17) $$\mathrm{layer-2}:\;\frac{\partial^{2}U}{\partial R^{2}}+\frac{1}{R}\frac{\partial U}{\partial R}\;=\varepsilon_{2}(P+\frac{U}{Da_{2}})\;(R_{1}<R<1).$$

The energy Equations (5), (6), (8), and (9) are rewritten as

(18) $$\mathrm{layer-1}:\;k_{s1}\left(\frac{\partial^{2}\theta_{s}}{\partial R^{2}}+\frac{1}{R}\frac{\partial \theta_{s}}{\partial R}\right)-Bi_{1}\left(\theta_{s}-\theta_{f}\right)=0\;(0<R<R_{1}),$$

(19) $$\mathrm{layer-2}:\;k_{s2}\left(\frac{\partial^{2}\theta_{s}}{\partial R^{2}}+\frac{1}{R}\frac{\partial \theta_{s}}{\partial R}\right)-Bi_{2}\left(\theta_{s}-\theta_{f}\right)=0\;(R_{1}<R<1),$$

(20) $$\mathrm{layer-1}:\;k_{1}\left(\frac{\partial^{2}\theta_{f}}{\partial R^{2}}+\frac{1}{R}\frac{\partial \theta_{f}}{\partial R}\right)+Bi_{1}\left(\theta_{s}-\theta_{f}\right)=2U\;(0<R<R_{1}),$$

(21) $$\mathrm{layer-2}:\;k_{2}\left(\frac{\partial^{2}\theta_{f}}{\partial R^{2}}+\frac{1}{R}\frac{\partial \theta_{f}}{\partial R}\right)+Bi_{2}\left(\theta_{s}-\theta_{f}\right)=2U\;(R_{1}<R<1).$$

Further, the boundary conditions are as follows,

(22) $$R=1:U=0\;\theta_{f}=\theta_{s}=0,$$

(23) $$R=0:\frac{\partial U}{\partial R}=0\frac{\partial \theta_{s}}{\partial R}=\frac{\partial \theta_{f}}{\partial R}=0,$$

(24) $$\begin{array}{c} R=R_{1}:U_{1}=U_{2}\;\frac{1}{\varepsilon_{1}}\frac{\partial U}{\partial R}=\frac{1}{\varepsilon_{2}}\frac{\partial U}{\partial R}\;\theta_{f1}=\theta_{f2}\;k_{1}\frac{\partial \theta_{f}}{\partial R}=k_{2}\frac{\partial \theta_{f}}{\partial R}\;\theta_{s1}=\theta_{s2}\\ k_{s1}\frac{\partial \theta_{s}}{\partial R}=k_{s2}\frac{\partial \theta_{s}}{\partial R} \end{array}$$

## 3. Analytical Solutions

Equations (16)–(21) are solved by using Modified Bessel Equation (MBE) [37]. The solutions are shown as follows.

### 3.1. Velocity Solutions

Combining Equations (16) and (17) with boundary conditions Equations (22)–(24), the velocity field is obtained as follows:

(25) $$U=\{\begin{array}{ll} P\left[C_{1}I_{0}\left(s_{1}R\right)+C_{2}K_{0}\left(s_{1}R\right)-Da_{1}\right] & \left(0<R<R_{1}\right)\\ P\left[C_{3}I_{0}\left(s_{2}R\right)+C_{4}K_{0}\left(s_{2}R\right)-Da_{2}\right] & \left(R_{1}<R<1\right) \end{array},$$

where the functions $I_{0}(z)$ and $K_{0}(z)$ are the first and second kinds of zero order modified Bessel function.

(26) $$\frac{1}{A}\int_{A}UdA=1,$$

(27) $$P=\frac{1}{\left\{\frac{2C_{1}}{s_{1}}I_{1}\left(s_{1}R_{1}\right)R_{1}-DaR_{1}^{2}+\frac{2C_{3}}{s_{2}}\left[I_{1}\left(s_{2}\right)-I_{1}\left(s_{2}R_{1}\right)R_{1}\right]-\frac{2C_{4}}{s_{2}}\left[K_{1}\left(s_{2}\right)-K_{1}\left(s_{2}R_{1}\right)R_{1}\right]-Da_{2}\left(1-R_{1}^{2}\right)\right\}}$$

where the constants $C_{1}$, $C_{2}$, $C_{3}$, and $C_{4}$ are derived as follows:

(28) $$C_{1}=A-BC_{4},$$

(29) $$C_{2}=0,$$

(30) $$C_{3}=\frac{Da_{2}-C_{4}K_{0}\left(s_{2}\right)}{I_{0}\left(s_{2}\right)}$$

(31) $$C_{4}=\frac{AI_{0}\left(s_{1}R_{1}\right)-Da_{1}+Da_{2}-\frac{I_{0}\left(s_{2}R_{1}\right)}{I_{0}\left(s_{2}\right)}Da_{2}}{\frac{K_{0}\left(s_{2}R_{1}\right)I_{0}\left(s_{2}\right)-K_{0}\left(s_{2}\right)I_{0}\left(s_{2}R_{1}\right)}{I_{0}\left(s_{2}\right)}+BI_{0}\left(s_{1}R_{1}\right)},$$

where $A=\frac{\varepsilon_{1}s_{2}}{\varepsilon_{2}s_{1}}\cdot \frac{1}{I_{1}\left(s_{1}R_{1}\right)}\cdot \frac{I_{1}\left(s_{2}R_{1}\right)}{I_{0}\left(s_{2}\right)}Da_{2}$, and $B=\frac{\varepsilon_{1}s_{2}}{\varepsilon_{2}s_{1}}\cdot \frac{1}{I_{1}\left(s_{1}R_{1}\right)}\cdot \frac{K_{0}\left(s_{2}\right)I_{1}\left(s_{2}R_{1}\right)+K_{1}\left(s_{2}R_{1}\right)I_{0}\left(s_{2}\right)}{I_{0}\left(s_{2}\right)}$.

### 3.2. Temperature Distribution

The analytical solutions for temperature distributions are derived as follows:

*layer-1* $\left(0<R<R_{1}\right)$

(32) $$\theta_{s}=\frac{P}{k_{s1}+k_{1}}\left[\begin{array}{l} D_{1}+D_{2}InR+D_{3}I_{0}\left(t_{1}R\right)+D_{4}K_{0}\left(t_{1}R\right)-\\ \frac{2t_{1}^{2}}{s_{1}^{2}\left(s_{1}^{2}-t_{1}^{2}\right)}C_{1}I_{0}\left(s_{1}R\right)-\frac{Da_{1}}{2}\left(R^{2}+\frac{4}{t_{1}^{2}}\right) \end{array}\right],$$

(33) $$\theta_{f}=\theta_{s}-\frac{P}{k_{1}}\left[D_{3}I_{0}\left(t_{1}R\right)+D_{4}K_{0}\left(t_{1}R\right)-\frac{2}{s_{1}^{2}-t_{1}^{2}}C_{1}I_{0}\left(s_{1}R\right)-\frac{2Da_{1}}{t_{1}^{2}}\right],$$

*layer-2*$\left(R_{1}<R<1\right)$

(34) $$\theta_{s}=\frac{P}{k_{s2}+k_{2}}\left\{\begin{array}{l} D_{5}+D_{6}InR+D_{7}I_{0}\left(t_{2}R\right)+D_{8}K_{0}\left(t_{2}R\right)-\\ \frac{2t_{2}^{2}}{s_{2}^{2}\left(s_{2}^{2}-t_{2}^{2}\right)}\left[C_{3}I_{0}\left(s_{2}R\right)+C_{4}K_{0}\left(s_{2}R\right)\right]-\frac{Da_{2}}{2}\left(R^{2}+\frac{4}{t_{2}^{2}}\right) \end{array}\right\},$$

(35) $$\theta_{f}=\theta_{s}-\frac{P}{k_{2}}\left\{D_{7}I_{0}\left(t_{2}R\right)+D_{8}K_{0}\left(t_{2}R\right)-\frac{2}{s_{2}^{2}-t_{2}^{2}}\left[C_{3}I_{0}\left(s_{2}R\right)+C_{4}K_{0}\left(s_{2}R\right)\right]-\frac{2Da_{2}}{t_{2}}\right\},$$

where the constants $D_{1}$–$D_{8}$ are as follows:

(36) $$D_{1}=w_{7}D_{8}+w_{8},$$

(37) $$D_{2}=0,$$

(38) $$D_{3}=w_{2}D_{8}+w_{3},$$

(39) $$D_{4}=0,$$

(40) $$D_{5}=w_{11},$$

(41) $$D_{6}=\frac{w_{5}D_{8}+w_{6}}{w_{4}},$$

(42) $$D_{7}=\frac{w_{1}-D_{8}K_{0}\left(t_{2}\right)}{I_{0}\left(t_{2}\right)},$$

(43) $$D_{8}=w_{10}/w_{9}.$$

The constants $w_{1}$–$w_{11}$ are represented as follows:

(44) $$w_{1}=\frac{2}{s_{2}^{2}-t_{2}^{2}}\left[C_{3}I_{0}\left(s_{2}\right)+C_{4}K_{0}\left(s_{2}\right)\right]+\frac{2Da_{2}}{t_{2}^{2}},$$

(45) $$w_{2}=\frac{k_{1}}{k_{2}}\frac{I_{0}\left(t_{2}\right)K_{0}\left(t_{2}R_{1}\right)-I_{0}\left(t_{2}R_{1}\right)K_{0}\left(t_{2}\right)}{I_{0}\left(t_{2}\right)I_{0}\left(t_{1}R_{1}\right)},$$

(46) $$\begin{array}{ll} w_{3} & =\frac{k_{1}}{k_{2}I_{0}\left(t_{1}R_{1}\right)}\left\{\frac{I_{0}\left(t_{2}R_{1}\right)}{I_{0}\left(t_{2}\right)}w_{1}-\frac{2Da_{2}}{t_{2}^{2}}-\frac{2}{s_{2}^{2}-t_{2}^{2}}\left[C_{3}I_{0}\left(s_{2}R_{1}\right)+C_{4}K_{0}\left(s_{2}R_{1}\right)\right]\right\}\\ & +\frac{2C_{1}I_{0}\left(s_{1}R_{1}\right)}{\left(s_{1}^{2}-t_{1}^{2}\right)I_{0}\left(t_{1}R_{1}\right)}+\frac{2Da_{1}}{t_{1}^{2}I_{0}\left(t_{1}R_{1}\right)}, \end{array}$$

(47) $$w_{4}=\frac{k_{s2}}{\left(k_{2}+k_{s2}\right)R_{1}},$$

(48) $$w_{5}=\frac{k_{s1}t_{1}I_{1}\left(t_{1}R_{1}\right)w_{2}}{k_{s1}+k_{1}}+\frac{k_{s2}t_{2}}{k_{s2}+k_{2}}\cdot \frac{K_{0}\left(t_{2}\right)I_{1}\left(t_{2}R_{1}\right)+K_{1}\left(t_{2}R_{1}\right)I_{0}\left(t_{2}\right)}{I_{0}\left(t_{2}\right)},$$

(49) $$\begin{array}{ll} w_{6} & =\frac{k_{s1}t_{1}I_{1}\left(t_{1}R_{1}\right)w_{3}}{k_{s1}+k_{1}}-\frac{k_{s1}}{k_{s1}+k_{1}}\left[\frac{2t_{1}^{2}}{s_{1}\left(s_{1}^{2}-t_{1}^{2}\right)}C_{1}I_{1}\left(s_{1}R_{1}\right)+Da_{1}R_{1}\right]\\ & -\frac{k_{s2}}{k_{s2}+k_{2}}\cdot \{\frac{I_{1}\left(t_{2}R_{1}\right)w_{1}t_{2}}{I_{0}\left(t_{2}\right)}-\frac{2t_{2}^{2}}{s_{2}\left(s_{2}^{2}-t_{2}^{2}\right)}\left[C_{3}I_{1}\left(s_{1}R_{1}\right)-C_{4}K_{1}\left(s_{2}R_{1}\right)\right]-Da_{2}R_{1}\}, \end{array}$$

(50) $$w_{7}=\frac{k_{1}+k_{s1}}{k_{2}+k_{s2}}\left[\frac{w_{5}InR_{1}}{w_{4}}+\frac{I_{0}\left(t_{2}\right)K_{0}\left(t_{2}R_{1}\right)-K_{0}\left(t_{2}\right)I_{0}\left(t_{2}R_{1}\right)}{I_{0}\left(t_{2}\right)}\right]-w_{2}I_{0}\left(t_{1}R_{1}\right),$$

(51) $$\begin{array}{ll} w_{8} & =\frac{k_{1}+k_{s1}}{k_{2}+k_{s2}}\left\{\begin{array}{l} w_{11}+\frac{w_{6}InR_{1}}{w_{4}}+\frac{I_{0}\left(t_{2}R_{1}\right)w_{1}}{I_{0}\left(t_{2}\right)}-\frac{2t_{2}^{2}}{s_{2}^{2}\left(s_{2}^{2}-t_{2}^{2}\right)}\left[C_{3}I_{0}\left(s_{2}R_{1}\right)+C_{4}K_{0}\left(s_{2}R_{1}\right)\right]\\ -\frac{Da_{2}}{2}\left(R_{1}^{2}+\frac{4}{t_{2}^{2}}\right) \end{array}\right\}\\ & -w_{3}I_{0}\left(t_{1}R_{1}\right)+\frac{2t_{1}^{2}}{s_{1}^{2}\left(s_{1}^{2}-t_{1}^{2}\right)}C_{1}I_{0}\left(s_{1}R_{1}\right)+\frac{Da_{1}}{2}(R_{1}^{2}+\frac{4}{t_{1}^{2}}), \end{array}$$

(52) $$w_{9}=\frac{k_{s2}t_{2}}{k_{2}+k_{s2}}\cdot \frac{K_{0}\left(t_{2}\right)I_{1}\left(t_{2}R_{1}\right)+I_{0}\left(t_{2}\right)K_{1}\left(t_{2}R_{1}\right)}{I_{0}\left(t_{2}\right)}+\frac{k_{2}}{k_{2}+k_{s2}}\cdot \frac{w_{5}}{w_{4}R_{1}}+\frac{k_{s1}t_{1}I_{1}\left(t_{1}R_{1}\right)w_{2}}{k_{1}+k_{s1}},$$

(53) $$\begin{array}{ll} w_{10} & =\frac{2s_{1}C_{1}I_{1}\left(s_{1}R_{1}\right)}{s_{1}^{2}-t_{1}^{2}}-\frac{k_{1}}{k_{1}+k_{s1}}\left[\frac{2t_{1}^{2}}{\left(s_{1}^{2}-t_{1}^{2}\right)s_{1}}C_{1}I_{1}\left(s_{1}R_{1}\right)+Da_{1}R_{1}\right]-\frac{k_{s1}t_{1}I_{1}\left(t_{1}R_{1}\right)w_{3}}{k_{1}+k_{s1}}\\ & -\frac{k_{2}}{k_{2}+k_{s2}}\{\frac{w_{6}}{w_{4}R_{1}}-\frac{2t_{2}^{2}}{s_{2}\left(s_{2}^{2}-t_{2}^{2}\right)}\left[C_{3}I_{1}\left(s_{2}R_{1}\right)-C_{4}K_{1}\left(s_{2}R_{1}\right)\right]-Da_{2}R_{1}\}\\ & -\frac{2s_{2}}{s_{2}^{2}-t_{2}^{2}}\left[C_{3}I_{1}\left(s_{2}R_{1}\right)-C_{4}K_{1}\left(s_{2}R_{1}\right)\right]+\frac{k_{s2}t_{2}I_{1}\left(t_{2}R_{1}\right)w_{1}}{\left(k_{2}+k_{s2}\right)I_{0}\left(t_{2}\right)}, \end{array}$$

(54) $$w_{11}=\frac{Da_{2}}{2}-\frac{2\left[C_{3}I_{0}\left(s_{2}\right)+C_{4}K_{0}\left(s_{2}\right)\right]}{s_{2}^{2}}.$$

### 3.3. Dimensionless Parameters

Based on the analytical solutions for velocity field and temperature distribution, the friction coefficient and the Nuesselt number are obtained as follows:

(55) f=−dp/dx⋅4r2ρfum2=−8PDa⋅Re,

(56) $$Nu=\frac{\bar{h}}{k_{f}}\cdot 2R=\frac{2Rq_{w}}{k_{f}(T_{w}-T_{f.b})}=-\frac{2}{k_{0}\cdot \theta_{f.b}},$$

where $\theta_{f,b}$ can be deduced by Equation (57). The detail formula for $\theta_{f,b}$ is presented in the Appendix A.

(57) $$\theta_{f,b}=\frac{\frac{1}{A}\int_{A}U\theta_{f}dA}{\frac{1}{A}\int_{A}UdA}=\frac{1}{\pi }\int_{0}^{2\pi }\int_{0}^{1}U\theta_{f}RdRd\varphi =2\int_{0}^{R_{1}}U\theta_{f}RdR+2\int_{R_{1}}^{1}U\theta_{f}RdR.$$

### 3.4. Entropy Generation Rate

The irreversibility of the studied system can be related to two effects: heat transfer across a finite (nonzero) temperature difference, as well as fluid friction [38]. Based on the equation mentioned above, the detailed derivations of the local entropy generation rates within two phases of porous media are given as follows [14], respectively:

*layer-1:*

(58) $$S_{f1}^{\prime \prime \prime }=\frac{k_{fe1}}{T_{f1}^{2}}\left[{\left(\frac{\partial T_{f1}}{\partial x}\right)}^{2}+{\left(\frac{\partial T_{f1}}{\partial r}\right)}^{2}\right]+\frac{h_{1}a_{1}{\left(T_{s1}-T_{f1}\right)}^{2}}{T_{s1}T_{f1}}+\frac{\mu_{f}u_{f1}^{2}}{K_{1}T_{f1}}+\frac{\mu_{eff}}{T_{f1}}{\left(\frac{\partial u_{f1}}{\partial r}\right)}^{2}\;(0<r<r_{1})$$

(59) $$S_{s1}^{\prime \prime \prime }=\frac{k_{se1}}{T_{s1}^{2}}\left[{\left(\frac{\partial T_{s1}}{\partial x}\right)}^{2}+{\left(\frac{\partial T_{s1}}{\partial r}\right)}^{2}\right]+\frac{h_{1}a_{1}{\left(T_{s1}-T_{f1}\right)}^{2}}{T_{s1}T_{f1}}\;(0<r<r_{1}),$$

*layer-2:*

(60) $$S_{f2}^{\prime \prime \prime }=\frac{k_{fe2}}{T_{f2}^{2}}\left[{\left(\frac{\partial T_{f2}}{\partial x}\right)}^{2}+{\left(\frac{\partial T_{f2}}{\partial r}\right)}^{2}\right]+\frac{h_{2}a_{2}{\left(T_{s2}-T_{f2}\right)}^{2}}{T_{s2}T_{f2}}+\frac{\mu_{f}u_{f2}^{2}}{K_{2}T_{f2}}+\frac{\mu_{eff}}{T_{f2}}{\left(\frac{\partial u_{f2}}{\partial r}\right)}^{2}\;(r_{1}<r<r_{2})$$

(61) $$S_{s2}^{\prime \prime \prime }=\frac{k_{se2}}{T_{s2}^{2}}\left[{\left(\frac{\partial T_{s2}}{\partial x}\right)}^{2}+{\left(\frac{\partial T_{s2}}{\partial r}\right)}^{2}\right]+\frac{h_{2}a_{2}{\left(T_{s2}-T_{f2}\right)}^{2}}{T_{s2}T_{f2}}\;(r_{1}<r<r_{2})$$

In terms of the dimensionless parameters shown in Equation (15), the dimensionless local entropy generation rates are defined,

*layer-1:*

(62) $$\begin{array}{ll} N_{f1}^{\prime \prime \prime } & =\frac{S_{f1}^{\prime \prime \prime }\cdot r_{2}^{2}}{k_{se1}}=\frac{k_{1}\left[{\left(\frac{\partial \theta_{f1}}{\partial R}\right)}^{2}+\frac{1}{Pe^{2}}\right]}{{\left(\theta_{f1}+F\right)}^{2}}+\frac{Bi_{1}{\left(\theta_{s1}-\theta_{f1}\right)}^{2}}{\left(\theta_{f1}+F)(\theta_{s1}+F\right)}\\ & +\frac{Br\cdot U_{f1}^{2}}{Da_{1}(\theta_{f1}+F)}+\frac{Br}{\varepsilon_{1}(\theta_{f1}+F)}{\left(\frac{\partial U_{f1}}{\partial R}\right)}^{2} \end{array}\left(0<R<R_{1}\right)\;,$$

(63) $$N_{s1}^{\prime \prime \prime }=\frac{S_{s1}^{\prime \prime \prime }\cdot r_{2}^{2}}{k_{se1}}=\frac{\left[{\left(\frac{\partial \theta_{s1}}{\partial R}\right)}^{2}+\frac{1}{Pe^{2}}\right]}{{\left(\theta_{s1}+F\right)}^{2}}+\frac{Bi_{1}{\left(\theta_{s1}-\theta_{f1}\right)}^{2}}{\left(\theta_{f1}+F\right)\left(\theta_{s1}+F\right)}\;\left(0<R<R_{1}\right),$$

*layer-2:*

(64) $$\begin{array}{ll} N_{f2}^{\prime \prime \prime } & =\frac{S_{f2}^{\prime \prime \prime }\cdot r_{2}^{2}}{k_{se1}}=\frac{k_{2}\left[{\left(\frac{\partial \theta_{f2}}{\partial R}\right)}^{2}+\frac{1}{Pe^{2}}\right]}{{\left(\theta_{f2}+F\right)}^{2}}+\frac{Bi_{2}{\left(\theta_{s2}-\theta_{f2}\right)}^{2}}{\left(\theta_{f2}+F)(\theta_{s2}+F\right)}\\ & +\frac{Br\cdot U_{f2}^{2}}{Da_{2}(\theta_{f2}+F)}+\frac{Br}{\varepsilon_{2}(\theta_{f2}+F)}{\left(\frac{\partial U_{f2}}{\partial R}\right)}^{2} \end{array}\left(R_{1}<R<1\right),$$

(65) $$N_{s2}^{\prime \prime \prime }=\frac{S_{s2}^{\prime \prime \prime }\cdot r_{2}^{2}}{k_{se1}}=\frac{k_{s2}\left[{\left(\frac{\partial \theta_{s2}}{\partial R}\right)}^{2}+\frac{1}{Pe^{2}}\right]}{{\left(\theta_{s2}+F\right)}^{2}}+\frac{Bi_{2}{\left(\theta_{s2}-\theta_{f2}\right)}^{2}}{\left(\theta_{f2}+F\right)\left(\theta_{s2}+F\right)}\;\left(R_{1}<R<1\right).$$

Accordingly, the dimensionless total entropy generation rate for the tube can be calculated using Equation (66).

(66) $$N_{t}=2\pi \left[\int_{0}^{R_{1}}\left(N_{f1}^{\prime \prime \prime }+N_{s1}^{\prime \prime \prime }\right)RdR+\int_{R_{1}}^{1}(N_{f2}^{\prime \prime \prime }+N_{s2}^{\prime \prime \prime })RdR\right].$$

## 4. Results and Discussion

### 4.1. Validation of Solutions

**Figure 2:**
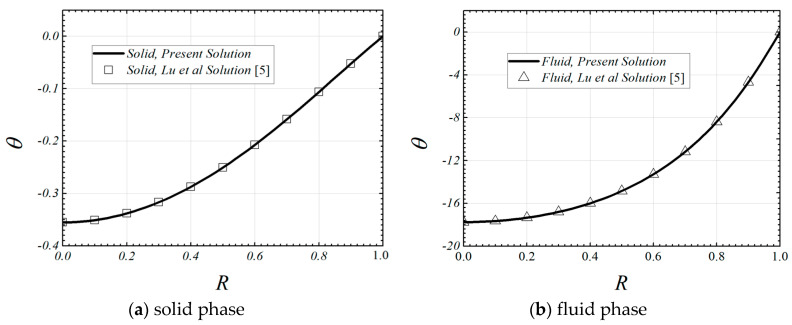
Temperature distributions for the fluid and the solid phases of the present study and Reference [5]. ($\varepsilon_{1}=\varepsilon_{2}=0.9,\;Da_{1}=10^{-3},\;Da_{2}=10^{-3},Bi_{1}=Bi_{2}=0.1,k_{1}=k_{2}=0.01,R_{1}=0.5$).

The analytical solutions in this paper can be validated for a limiting case in which the tube is fully filled with single layer porous medium. By setting the same pertinent parameters for the two porous layers, the present temperature distributions are exactly the same as those of the previous work of Lu et al. [5], as shown in Figure 2. When $\varepsilon_{1}=\varepsilon_{2}=1$ and the Darcy number approaches infinity, the present solution of the Nusselt number is 4.365, which is very close to the classical theoretical and experimental value for thermally fully developed clear flow. By setting $Bi_{1}=Bi_{2}=10,000$, and the pertinent parameters is the same as those of Reference [39], the present solutions of the thermally fully developed Nusselt number are almost the same as the previous numerical results obtained by Pavel and Mohamad [39] under the LTE condition, as shown in Table 1. Furthermore, the comparison between the Nusselt number of present study and the experimental and numerical results obtained by Pavel and Mohamad [39] is shown in Table 2. It is found that the Nusselt number of present study is less than the experimental and numerical values. The reason is as follows: the Nusselt number of present study is for the thermally fully developed flow; however, both the experimental and numerical results of the [39] are the average Nusselt number for the entire tube, including the thermally developing section and the thermally fully developed section.

### 4.2. Velocity Distribution

**Figure 3:**
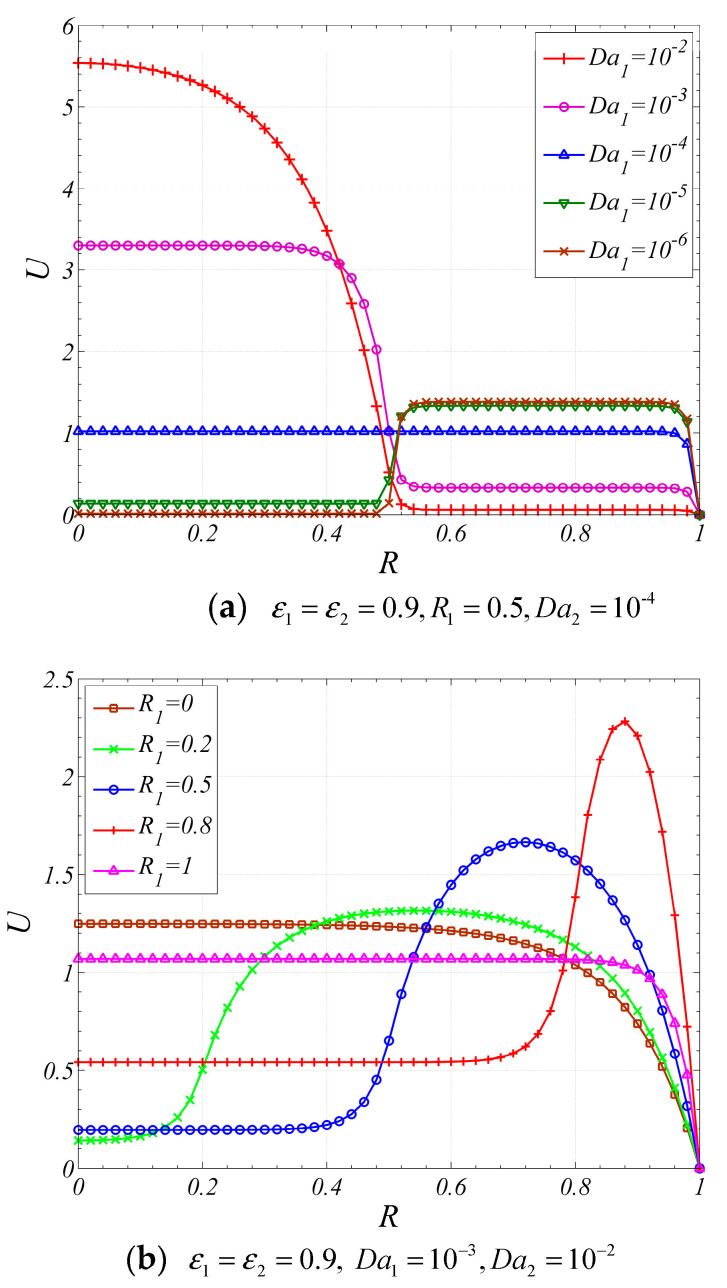
Effects of pertinent parameters on velocity distribution.

The influence of $Da_{1}$ on velocity profile is indicated in Figure 3a. As shown in the figure, the velocity profiles changes suddenly at the interface due to the sudden change of permeability across the interface. It is also found that the peak value of velocity appears in the layer with larger Da. In addition, when the Da difference between two porous layers is large enough, the fluid within the layer with smaller Da is almost stationary. This is because smaller Da means lower permeability for the fluid flow. Therefore, the fluid tends to flow through the layer with larger Da, while only a small portion flows through the other layer at a lower velocity.

Figure 3b shows the velocity distribution for different dimensionless interfacial radius. As shown in the figure, for the case of when $Da_{1}<Da_{2}$ and $R_{1}<0.8$, the peak value of velocity increases and its location shifts towards the wall as dimensionless interfacial radius increases.

### 4.3. Temperature Distribution

**Figure 4:**
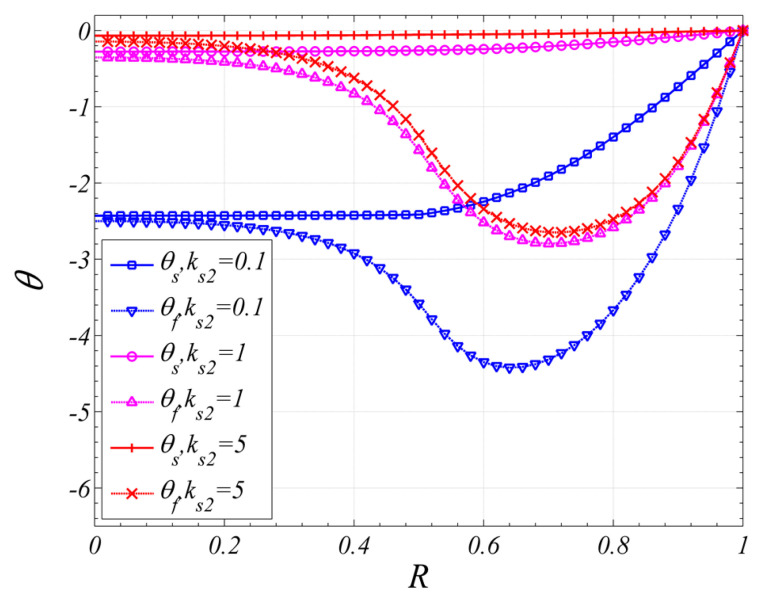
The effect of thermal conductivity ratio of solid on temperature distribution ($\varepsilon_{1}=\varepsilon_{2}=0.9,\;Da_{1}=10^{-5},\;Da_{2}=10^{-3},Bi_{1}=Bi_{2}=1,k_{1}=k_{2}=0.01,R_{1}=0.5$).

The effect of $k_{s2}$ on the temperature distributions is shown in Figure 4. As shown in the figure, both the temperatures of two phases increase with the increase of $k_{s2}$.

**Figure 5:**
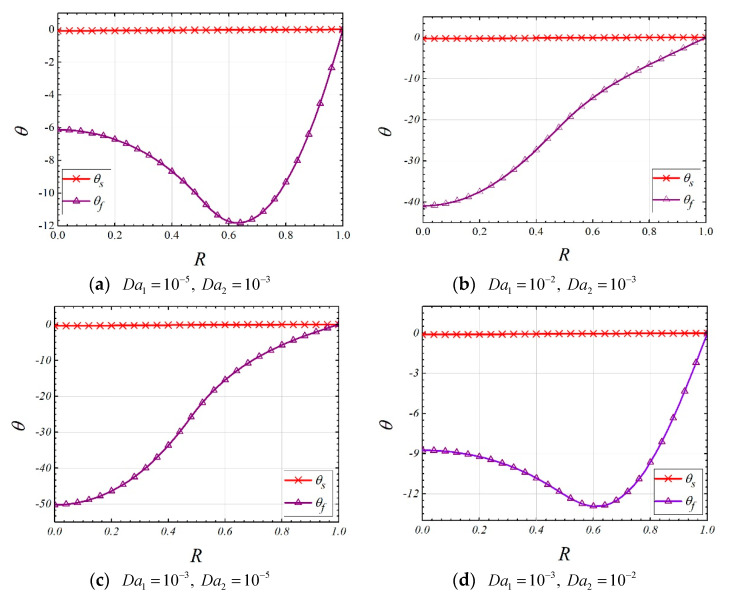
The effects of $Da_{1}$ and $Da_{2}$ on the temperature distribution ($\varepsilon_{1}=\varepsilon_{2}=0.9,\;Bi_{1}=Bi_{2}=0.1,k_{1}=k_{2}=0.01,k_{s2}=1,R_{1}=0.5$).

The effects of $Da_{1}$ and $Da_{2}$ on the temperature distributions are shown in Figure 5. It is found that both increasing $Da_{2}$ and decreasing $Da_{1}$ can lead to more uniform temperature distribution of fluid phase. This is because, when large $Da_{2}$ or small $Da_{1}$ is employed, the majority of the fluid will flow through the *layer-2*, which is closer to heated wall. Therefore, more heat is transferred to fluid phase within the *layer-2*, and a small amount of energy is transferred into *layer-1*, which results in a small temperature difference within *layer-1*.

### 4.4. Heat Transfer Performance

**Figure 6:**
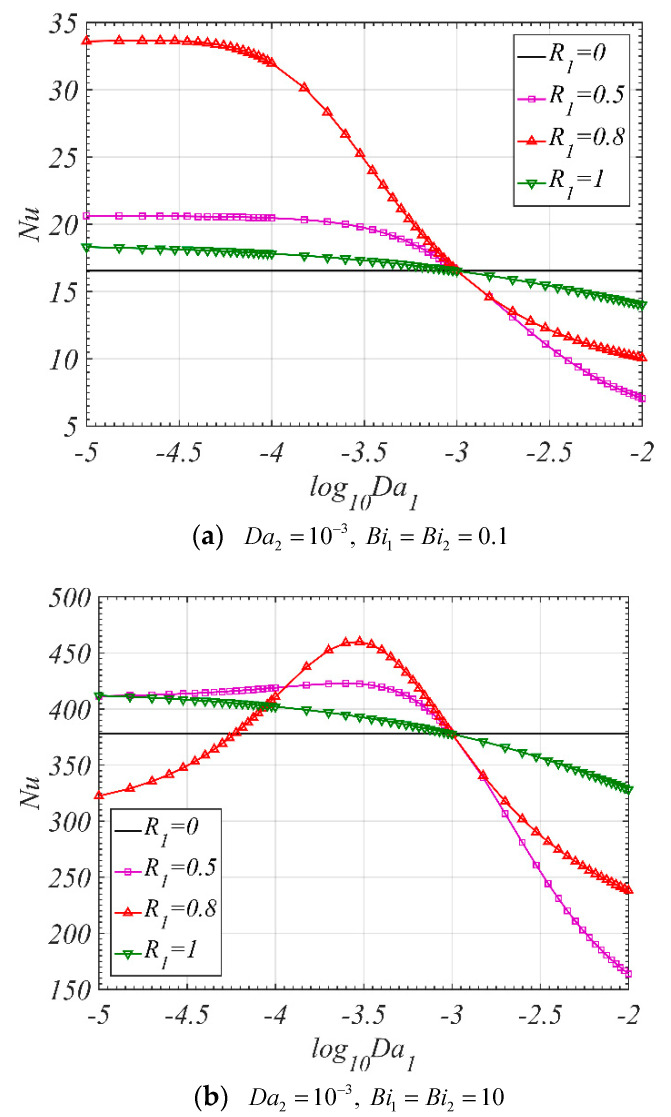
The effect of $Da_{1}$ on Nu ($\varepsilon_{1}=\varepsilon_{2}=0.9,\;k_{1}=k_{2}=0.01,\;k_{s2}=1$).

Figure 6 shows the trend of Nu versus $Da_{1}$. When the Biot number is small, which means a weak internal heat transfer between the solid and fluid phases in the porous media, the Nu decreases with the increase of the $Da_{1}$, as shown in Figure 6a. However, when the Biot number is large, the Nu increases firstly and then decreases gradually with the increase of the $Da_{1}$. As a result, a maximum value of Nu can be found in Figure 6b.

**Figure 7:**
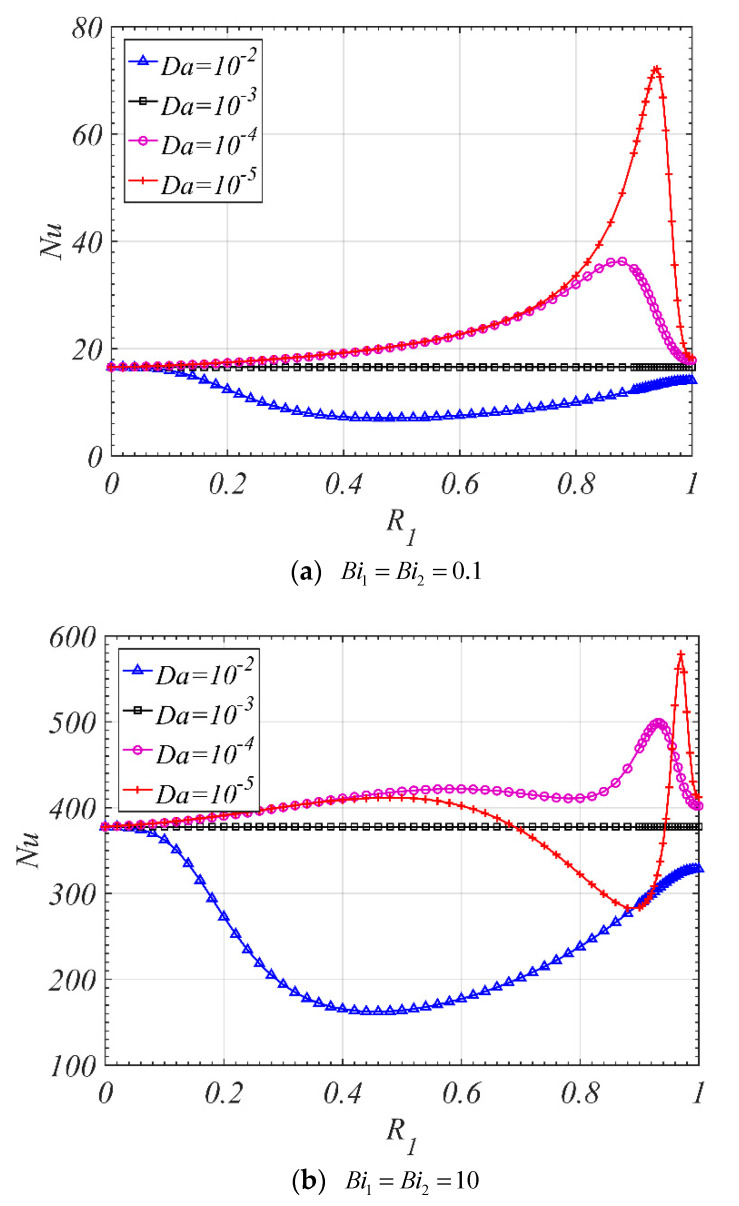
Nu varies with $R_{1}$ for different $Da_{1}$ ($\varepsilon_{1}=\varepsilon_{2}=0.9,\;k_{1}=k_{2}=0.01,k_{s2}=1,Da_{2}=10^{-3}$).

The effects of $R_{1}$ on Nu are shown in Figure 7. When $Da_{1}$ is larger than $Da_{2}$, the Nu decreases firstly and then increases with the increase of $R_{1}$, and a minimum value can be found, which is smaller than that for the tube fully filled with corresponding single layer porous medium ($R_{1}=0$, or $R_{1}=1$). However, when $Da_{1}$ is smaller than $Da_{2}$, there is one local maximum value for the Nu when the Biot number is small, as shown in Figure 7a; or there are two local maximum values for the Nu when the Biot number is large, as shown in Figure 7b. It can be found that, when $Da_{1}$ is smaller than $Da_{2}$, the Nu for the tube filled with double-layer porous media can be larger than that for the tube filled with corresponding single layer porous medium ($R_{1}=0$, or $R_{1}=1$). To the best of authors’ knowledge, the above-mentioned analysis of the Nu for the tube filled with double-layer porous media is presented for the first time in the literature.

**Figure 8:**
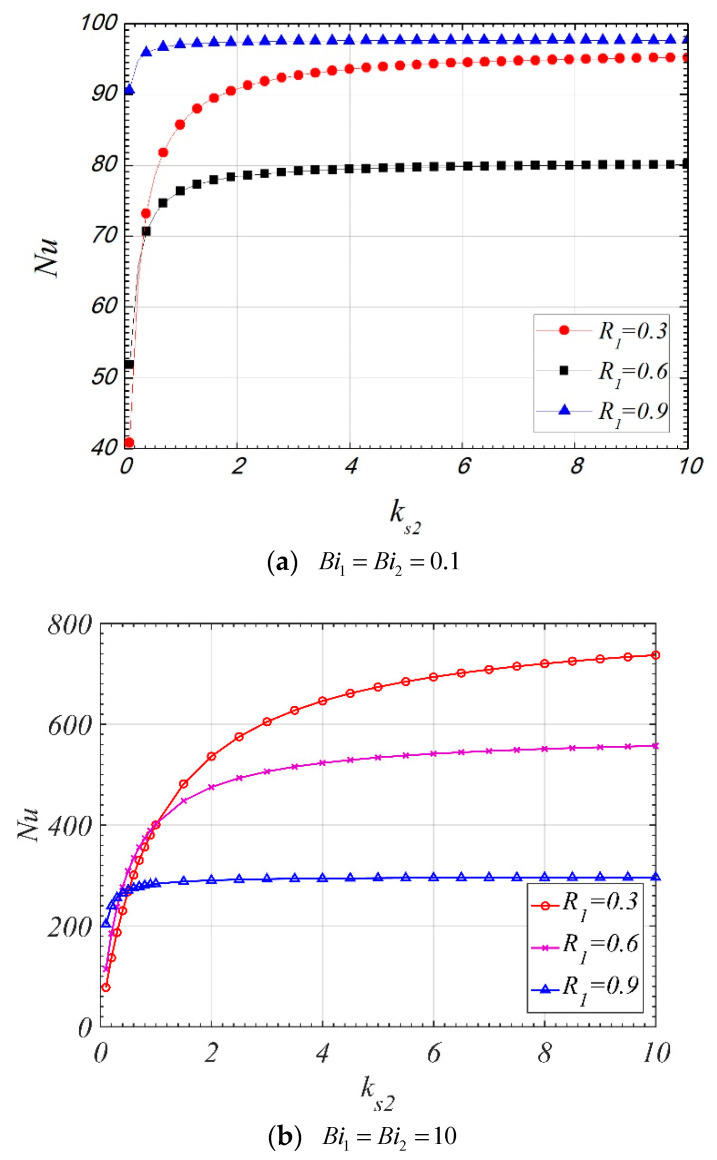
Nu varies with $k_{s2}$ ($\varepsilon_{1}=\varepsilon_{2}=0.9,\;k_{1}=k_{2}=0.01,Da_{1}=10^{-5},Da_{2}=10^{-3}$).

Figure 8 shows the trend of Nu versus $k_{s2}$. When $k_{s2}$ is less than one, the Nu increases dramatically with the increase of $k_{s2}$. However, the ascending tendency become less obviously when $k_{s2}>1$. This is because the value of the mean temperature of the fluid phase is negative, and it increases with the increase of $k_{s2}$, which is more obvious when $k_{s2}<1$, which can be found in Figure 4.

### 4.5. Local and Total Entropy Generation Rate

**Figure 9:**
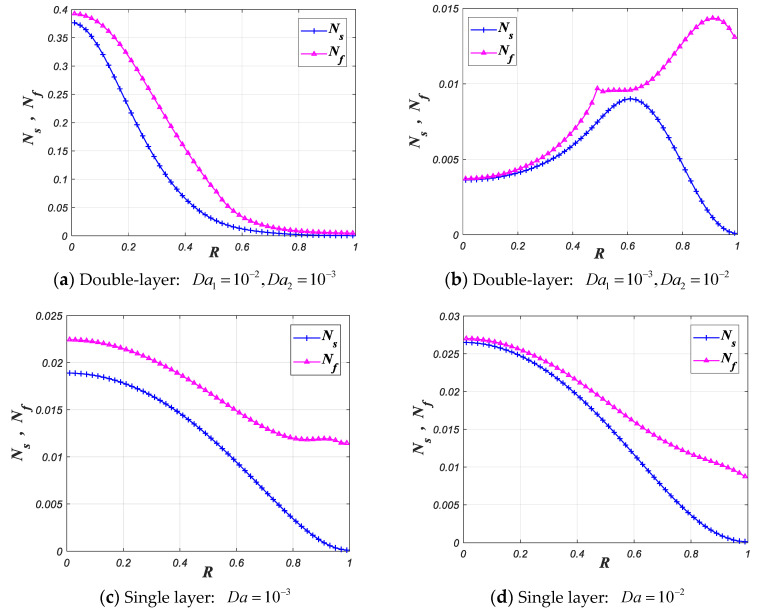
Local entropy generation rate varies with R for different Darcy number ($\varepsilon_{1}=\varepsilon_{2}=0.9,\;Bi_{1}=Bi_{2}=0.1,\;k_{1}=k_{2}=0.01,\;k_{s2}=1,\;F=50,\;Pe=10,\;Br=10^{-4},\;R_{1}=0.5$).

The local and total entropy generation rates are used to describe irreversibility of the tube filled with porous media in this work. Figure 9 shows the changes of the dimensionless local entropy generation rate for four filling methods, including: (a) filled with double-layer porous media and $Da_{1}>Da_{2}$, (b) filled with double-layer porous media and $Da_{1}<Da_{2}$, (c) filled with single layer porous medium with small Darcy number, and (d) filled with single layer porous medium with large Darcy number. Among the four filling methods, filling the tube with double-layer porous media with smaller Darcy number for *layer-1* (method b) can be more conducive to reduce the local entropy generation rate.

**Figure 10:**
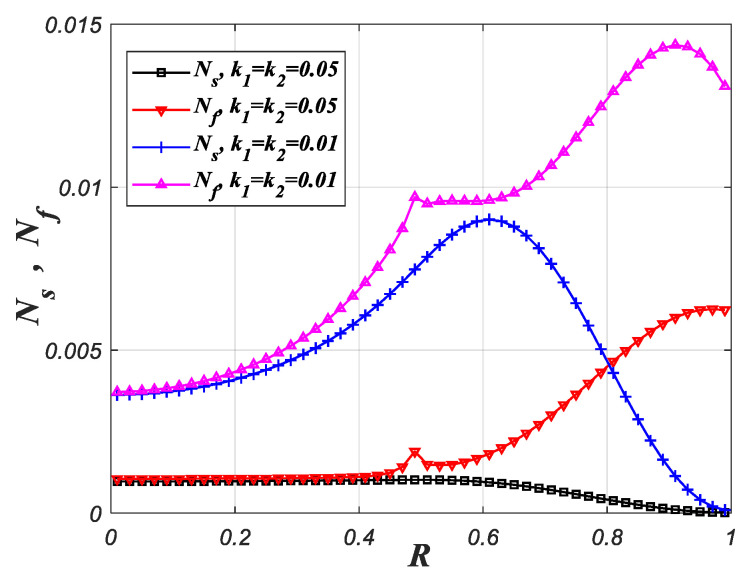
Local entropy generation rate varies with R for different $k_{1}$ and $k_{2}$ ($\varepsilon_{1}=\varepsilon_{2}=0.9,\;Da_{1}=10^{-3},\;Da_{2}=10^{-2},\;Bi_{1}=Bi_{2}=0.1,\;k_{s2}=1,\;F=50,\;Pe=10,\;Br=10^{-4},\;R_{1}=0.5$).

Figure 10 represents the impact of the $k_{1}$ and $k_{2}$ on the local entropy generation rate. As shown in Figure 10, increasing the $k_{1}$ and $k_{2}$ sharply decreases the values of local entropy generation rates. Increasing the $k_{1}$ and $k_{2}$ decreases the thermal conductivity resistance and the temperature difference between two phases in porous media, which reduces the entropy generation caused by the heat conduction within the fluid phase and the heat exchange between two phases in porous media simultaneously.

**Figure 11:**
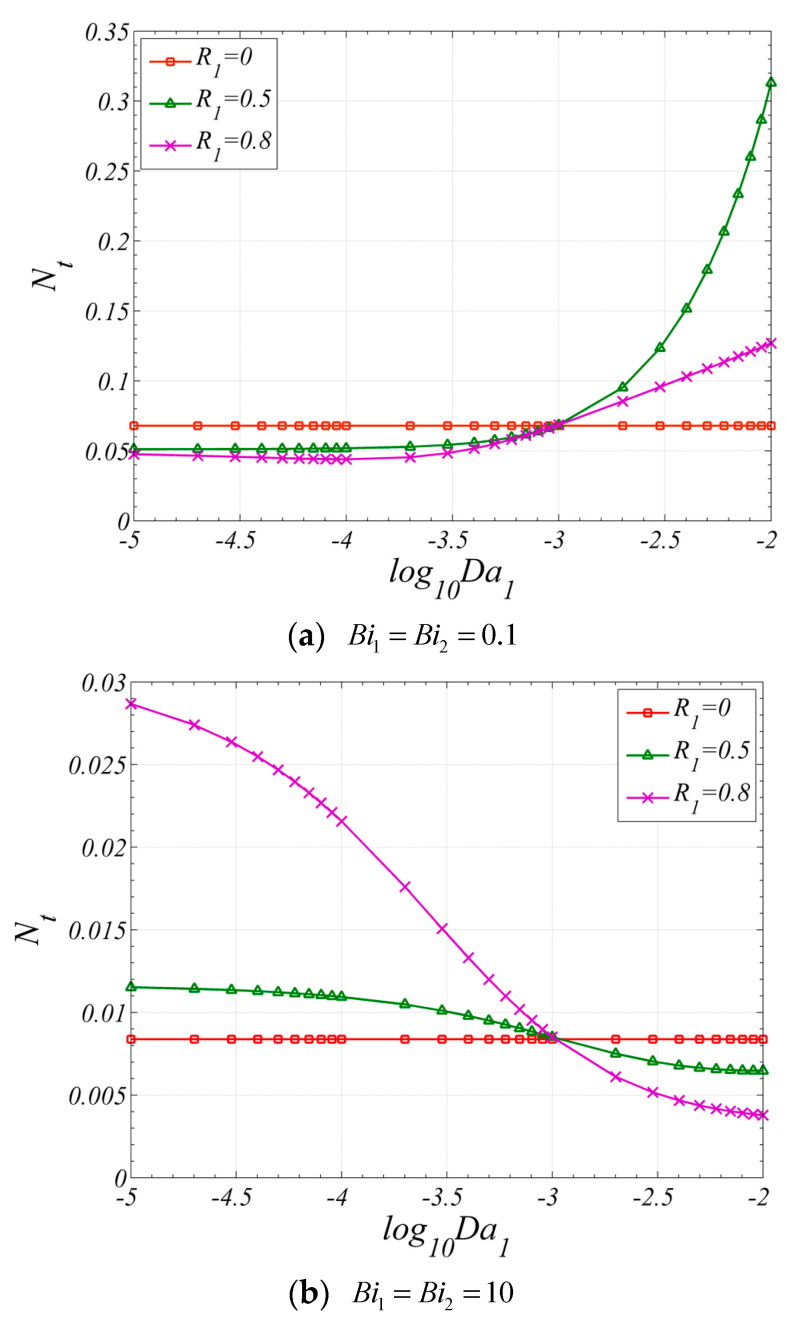
$N_{t}$ varies with $Da_{1}$ for different $R_{1}$. ($\varepsilon_{1}=\varepsilon_{2}=0.9,\;Da_{2}=10^{-3},\;k_{1}=k_{2}=0.01,\;k_{s2}=1,\;F=50,\;Pe=10,\;Br=10^{-4}$).

The effect of $Da_{1}$ on $N_{t}$ is shown in Figure 11. When the Biot number is small, as shown in Figure 11a, for the case of $R_{1}=0.5$, the $N_{t}$ increases with the increase of $Da_{1}$, for the case of $R_{1}=0.8$, the $N_{t}$ slightly decreases first and then increases with the increase of $Da_{1}$. Two effects will be induced by the increase of $Da_{1}$: (a) the temperature difference between two phases in porous media will increase with the increase of $Da_{1}$, as shown in Figure 5, which will decrease the Nu, as shown in Figure 7a, and increase the entropy generation caused by heat exchange between two phases in porous media; and (b) the entropy generation caused by fluid friction will decrease with the increase of permeability. As a result, a minimum value can be found when $R_{1}=0.8$. When $R_{1}=0.5$, the proportion of the entropy generation caused by fluid friction is relatively small. Hence, the $N_{t}$ varies monotonically with $Da_{1}$. When the Biot number is large, the proportion of the entropy generation caused by heat transfer is relatively small, and the entropy generation caused by fluid friction will decrease with the increase of $Da_{1}$. Therefore, $N_{t}$ will decrease with the increase of $Da_{1}$, as shown in Figure 11b.

**Figure 12:**
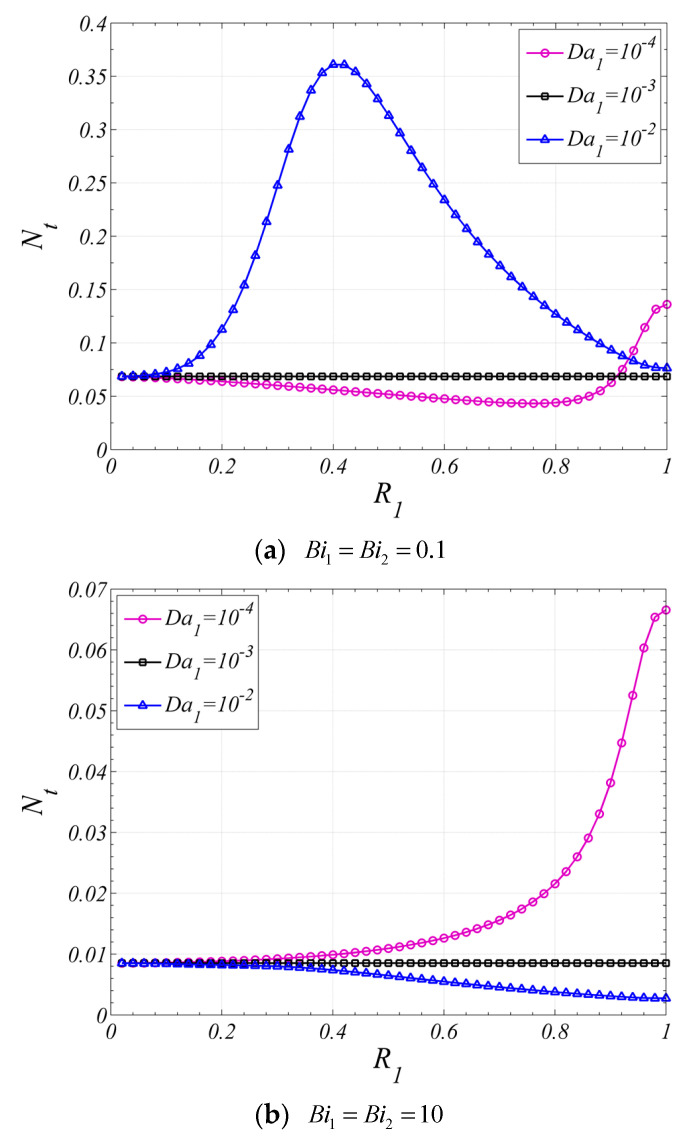
$N_{t}$ varies with $R_{1}$ for different ${Da}_{1}$ ($\varepsilon_{1}=\varepsilon_{2}=0.9,\;k_{1}=k_{2}=0.01,k_{s2}=1,Da_{2}=10^{-3}F=50,\;Pe=10,\;Br=10^{-4}$).

Figure 12 shows the influence of $R_{1}$ on the $N_{t}$. When the Biot number is small, both the effects of the heat transfer and fluid friction should be considered. When $Da_{1}<Da_{2}$, as the result of the Nu variation with $R_{1}$, which is shown in Figure 7a, the entropy generation caused by heat transfer will decrease firstly and then increase with the increase of $R_{1}$. In the meantime, the entropy generation caused by fluid friction will increase with the increase of $R_{1}$. Therefore, it can be found in Figure 12a that there is a minimum value for $N_{t}$ which is smaller than that for the tube fully filled with corresponding single layer porous medium ($R_{1}=0$, or $R_{1}=1$). When $Da_{1}>Da_{2}$, with the increase of $R_{1}$, the entropy generation caused by heat transfer will increase firstly and then decrease, and the entropy generation caused by fluid friction will decrease. Therefore, it can be found in Figure 12a that there is a maximum value for $N_{t}$.

However, when the Biot number is relatively large, as shown in Figure 12b, for the case of $Da_{1}=10^{-4}$, the entropy generation is mainly caused by fluid friction; therefore, the $N_{t}$ increases with the increase of $R_{1}$. For the case of $Da_{1}=10^{-2}$, the entropy generation caused by both heat transfer and fluid friction are much small, and the $N_{t}$ decreases with the increase of $R_{1}$.

**Figure 13:**
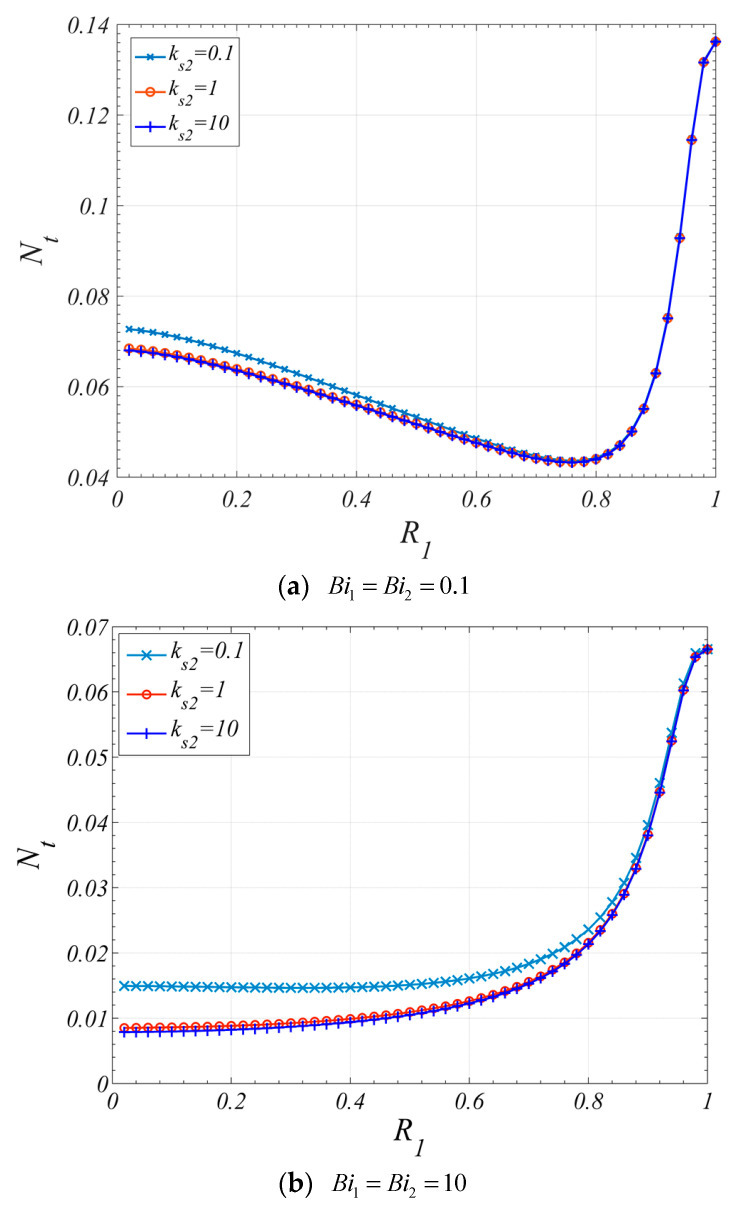
$N_{t}$ varies with $R_{1}$ for different $k_{s2}$ ($\varepsilon_{1}=\varepsilon_{2}=0.9,\;Da_{1}=10^{-4},\;Da_{2}=10^{-3},\;k_{1}=k_{2}=0.01,\;F=50,\;Pe=10,\;Br=10^{-4}$).

Figure 13 illustrates the influence of the $k_{s2}$ on $N_{t}$. It can be found that both increasing the Biot number and increasing the $k_{s2}$ can reduce the $N_{t}$. The influence of $k_{s2}$ on $N_{t}$ is negligible when $k_{s2}>1$. When the Biot number is small, as what have been mentioned above, a minimum value of $N_{t}$ can be found in Figure 13a. However, compared with Figure 7a and Figure 12a, it can be found that the dimensionless interfacial radius corresponding to the maximum value of the Nu is different from that corresponding to the minimum value of the $N_{t}$.

## 5. Conclusions

In this study, the forced convective heat transfer in a tube filled with double-layer porous media is analytically investigated. The Darcy-Brinkman equation and the LTNE model are employed for momentum and energy equations, respectively. The velocity field and temperature distributions are analytically solved, and the analytical solutions for Nusselt number, local, and total entropy generation rates are obtained. The analytical solutions are validated in the limiting case. Furthermore, the influences of the Darcy number, Biot number, thermal conductivity ratio, and dimensionless interfacial radius on flow and heat transfer, as well as irreversibility, are analyzed. The main conclusions are as follows:

(1) A more uniform temperature distribution of fluid phase within the tube filled with double-layer porous media can be obtained by decreasing $Da_{1}$ or increasing $Da_{2}$.
(2) When $Da_{1}$ is less than $Da_{2}$, the Nusselt number for the tube filled with double-layer porous media can be larger than that for the tube filled with corresponding single layer porous medium. However, when $Da_{1}$ is larger than $Da_{2}$, the Nusselt number for the tube filled with double-layer porous media can be less than that for the tube filled with corresponding single layer porous medium.
(3) When $Da_{1}$ is less than $Da_{2}$ and the Biot number is small, the total entropy generation rate for the tube filled with double-layer porous media can be less than that for the tube filled with corresponding single layer porous medium.
(4) When $Da_{1}$ is less than $Da_{2}$, the maximum value of the Nusselt number and the minimum value of the total entropy generation rate for the tube filled with double-layer porous media can be obtained by properly selecting the pertinent parameters, such as Darcy number, Biot number, and dimensionless interfacial radius. However, it should be noted that the dimensionless interfacial radius corresponding to the maximum value of the Nusselt number is different from that corresponding to the minimum value of the total entropy generation rate.

## Figures and Tables

**Table 1:** The thermally fully developed Nu number of present study when $Bi_{1}=Bi_{2}=10,000$ and Reference [39].

Da	$\frac{k_{fe}+k_{se}}{k_{f}}$	Nu (Present Study)	Nu ([39])
10^−2^	1.0	5.9812	5.9848
10^−3^	1.0	7.1380	7.1360
10^−4^	1.0	7.6957	7.6955
10^−5^	1.0	7.9002	7.9012

**Table 2:** The Nusselt number of the present study and the experimental and numerical average Nusselt number of Reference [39].

ε	Da	$\frac{k_{fe}+k_{se}}{k_{f}}$	Nu (Present Study When $Bi_{1}=Bi_{2}=10,000)$	Experimental Average Nu ([39])	Numerical Average Nu ([39])
0.993	$6.1786\times 10^{-4}$	1.06	7.7370	19.2204	21.8579
0.981	$1.9384\times 10^{-4}$	1.5	11.3711	29.6612	34.0109

## Nomenclature

a	interfacial area per unit volume of the porous media, m^−1^
Bi	Biot number
Br	Brinkman number
$c_{f}$	heat capacity of fluid, J·kg^−1^·K^−1^
Da	Darcy number
F	dimensionless wall temperature, defined in Equation (15)
h	heat transfer coefficient, W·m^−2^·K^−1^
K	permeability, m^2^
k	ratio of effective fluid thermal conductivity to that of solid
$k_{s}$	ratio of effective solid thermal conductivity of two layers
$N_{f}^{\prime \prime \prime }$	dimensionless local entropy generation rate within the fluid phase
$N_{s}^{\prime \prime \prime }$	dimensionless local entropy generation rate within the solid phase
$N_{t}$	dimensionless total entropy generation rate within the tube
Nu	overall Nusselt number
P	dimensionless pressure drop
Pe	Peclet number
p	pressure, N·m^−2^
R	dimensionless radius
r	radius, m
$S_{s}^{\prime \prime \prime }$	local entropy generation rate within the fluid phase
$S_{f}^{\prime \prime \prime }$	local entropy generation rate within the solid phase
T	temperature, K
tkr	thermal conductivity ratio, $k_{e}/k_{f}$
U	dimensionless velocity
u	velocity, m/s
Greek symbols	
ε	porosity
θ	dimensionless temperature
μ	dynamic viscosity, kg·m^−1^·s^−1^
ρ	density, kg·m^−3^
Subscripts	
b	bulk
e	effective
f	fluid phase
m	mass average
s	solid phase
w	wall
1	layer 1
2	layer 2

## Appendix A. $\theta_{f,b}=Q_{1}-Q_{2}+Q_{3}-Q_{4}$

$Q_{1}=\frac{2P^{2}}{k_{1}+k_{s1}}\left\{\begin{array}{l} \left[C_{1}D_{1}+\frac{2t_{1}^{2}Da_{1}C_{1}}{s_{1}^{2}(s_{1}^{2}-t_{1}^{2})}-\frac{2Da_{1}C_{1}}{t_{1}^{2}}\right]\times \frac{I_{1}(s_{1}R_{1})R_{1}}{s_{1}}+\\ C_{1}D_{3}\frac{I_{1}(s_{1}R_{1})I_{0}(t_{1}R_{1})s_{1}R_{1}-I_{0}(s_{1}R_{1})I_{1}(t_{1}R_{1})t_{1}R_{1}}{s_{1}^{2}-t_{1}^{2}}\\ -\frac{t_{1}^{2}C_{1}^{2}R_{1}^{2}}{s_{1}^{2}(s_{1}^{2}-t_{1}^{2})}\left[I_{0}^{2}(s_{1}R_{1})-I_{1}^{2}(s_{1}R_{1})\right]-\\ \frac{Da_{1}C_{1}}{2}\left[\frac{I_{1}(s_{1}R_{1})R_{1}^{3}}{s_{1}}-\frac{2I_{0}(s_{1}R_{1})R_{1}^{2}}{s_{1}^{2}}+\frac{4I_{1}(s_{1}R_{1})R_{1}}{s_{1}^{3}}\right]+\\ \left(\frac{2Da_{1}^{2}}{t_{1}^{2}}-Da_{1}D_{1}\right)\times \frac{R_{1}^{2}}{2}-Da_{1}D_{3}\times \frac{I_{1}(t_{1}R_{1})R_{1}}{t_{1}}+\frac{Da_{1}^{2}R_{1}^{4}}{8} \end{array}\right\}$
$Q_{2}=\frac{2P^{2}}{k_{1}}\left\{\begin{array}{l} C_{1}D_{3}\frac{I_{1}(s_{1}R_{1})I_{0}(t_{1}R_{1})s_{1}R_{1}-I_{0}(s_{1}R_{1})I_{1}(t_{1}R_{1})t_{1}R_{1}}{s_{1}^{2}-t_{1}^{2}}-\frac{C_{1}^{2}R_{1}^{2}}{s_{1}^{2}-t_{1}^{2}}\left[I_{0}^{2}(s_{1}R_{1})-I_{1}^{2}(s_{1}R_{1})\right]+\\ \left(\frac{2Da_{1}C_{1}}{s_{1}^{2}-t_{1}^{2}}-\frac{2Da_{1}C_{1}}{t_{1}^{2}}\right)\times \frac{I_{1}(s_{1}R_{1})R_{1}}{s_{1}}-Da_{1}D_{3}\times \frac{I_{1}(t_{1}R_{1})R_{1}}{t_{1}}+\frac{Da_{1}^{2}R_{1}^{2}}{t_{1}^{2}} \end{array}\right\}$
$Q_{3}=\frac{2P^{2}}{k_{2}+k_{s2}}\left\{\begin{array}{l} C_{3}D_{5}\frac{I_{1}(s_{2})-I_{1}(s_{2}R_{1})R_{1}}{s_{2}}+C_{3}D_{6}\frac{I_{0}(s_{2}R_{1})-I_{0}(s_{2})-I_{1}(s_{2}R_{1})InR_{1}s_{2}R_{1}}{s_{2}^{2}}+\\ C_{3}D_{7}\frac{I_{1}(s_{2})I_{0}(t_{2})s_{2}-I_{0}(s_{2})I_{1}(t_{2})t_{2}-I_{1}(s_{2}R_{1})I_{0}(t_{2}R_{1})s_{2}R_{1}+I_{0}(s_{2}R_{1})I_{1}(t_{2}R_{1})t_{2}R_{1}}{s_{2}^{2}-t_{2}^{2}}+\\ C_{3}D_{8}\frac{I_{1}(s_{2})K_{0}(t_{2})s_{2}+I_{0}(s_{2})K_{1}(t_{2})t_{2}-I_{1}(s_{2}R_{1})K_{0}(t_{2}R_{1})s_{2}R_{1}-I_{0}(s_{2}R_{1})K_{1}(t_{2}R_{1})t_{2}R_{1}}{s_{2}^{2}-t_{2}^{2}}-\\ \frac{Da_{2}C_{3}}{2}\left[\frac{I_{1}(s_{2})-I_{1}(s_{2}R_{1})R_{1}^{3}}{s_{2}}-\frac{2I_{0}(s_{2})-2I_{0}(s_{2}R_{1})R_{1}^{2}}{s_{2}^{2}}+\frac{4I_{1}(s_{2})-4I_{1}(s_{2}R_{1})R_{1}}{s_{2}^{3}}\right]-\\ \frac{2Da_{2}C_{3}}{t_{2}^{2}}\times \frac{I_{1}(s_{2})-I_{1}(s_{2}R_{1})R_{1}}{s_{2}}-C_{4}D_{5}\frac{K_{1}(s_{2})-K_{1}(s_{2}R_{1})R_{1}}{s_{2}}-\\ C_{4}D_{6}\frac{K_{0}(s_{2})-K_{0}(s_{2}R_{1})-K_{1}(s_{2}R_{1})InR_{1}s_{2}R_{1}}{s_{2}^{2}}+\\ C_{4}D_{7}\frac{I_{1}(t_{2})K_{0}(s_{2})t_{2}+I_{0}(t_{2})K_{1}(s_{2})s_{2}-I_{1}(t_{2}R_{1})K_{0}(s_{2}R_{1})t_{2}R_{1}-I_{0}(t_{2}R_{1})K_{1}(s_{2}R_{1})s_{2}R_{1}}{t_{2}^{2}-s_{2}^{2}}+\\ C_{4}D_{8}\frac{K_{1}(t_{2})K_{0}(s_{2})t_{2}-K_{0}(t_{2})K_{1}(s_{2})s_{2}-K_{1}(t_{2}R_{1})K_{0}(s_{2}R_{1})t_{2}R_{1}+K_{0}(t_{2}R_{1})K_{1}(s_{2}R_{1})s_{2}R_{1}}{t_{2}^{2}-s_{2}^{2}}+\\ \frac{Da_{2}C_{4}}{2}\left[\frac{K_{1}(s_{2})-K_{1}(s_{2}R_{1})R_{1}^{3}}{s_{2}}+\frac{2K_{0}(s_{2})-2K_{0}(s_{2}R_{1})R_{1}^{2}}{s_{2}^{2}}+\frac{4K_{1}(s_{2})-4K_{1}(s_{2}R_{1})R_{1}}{s_{2}^{3}}\right]+\\ \frac{2Da_{2}C_{4}}{t_{2}^{2}}\times \frac{K_{1}(s_{2})-K_{1}(s_{2}R_{1})R_{1}}{s_{2}}-\frac{2t_{2}^{2}C_{3}^{2}}{s_{2}^{2}(s_{2}^{2}-t_{2}^{2})}\left[\frac{I_{0}^{2}(s_{2})-I_{1}^{2}(s_{2})}{2}-\frac{I_{0}^{2}(s_{2}R_{1})-I_{1}^{2}(s_{2}R_{1})}{2}R_{1}^{2}\right]-\\ \frac{4t_{2}^{2}C_{3}C_{4}}{s_{2}^{2}(s_{2}^{2}-t_{2}^{2})}\left[\frac{I_{0}(s_{2})K_{0}(s_{2})+I_{1}(s_{2})K_{1}(s_{2})}{2}-\frac{I_{0}(s_{2}R_{1})K_{0}(s_{2}R_{1})+I_{1}(s_{2}R_{1})K_{1}(s_{2}R_{1})}{2}R_{1}^{2}\right]-\\ \frac{2t_{2}^{2}C_{4}^{2}}{s_{2}^{2}(s_{2}^{2}-t_{2}^{2})}\left[\frac{K_{0}^{2}(s_{2})-K_{1}^{2}(s_{2})}{2}-\frac{K_{0}^{2}(s_{2}R_{1})-K_{1}^{2}(s_{2}R_{1})}{2}R_{1}^{2}\right]-Da_{2}D_{5}\frac{1-R_{1}^{2}}{2}+\frac{Da_{2}^{2}(1-R_{1}^{4})}{8}-\\ Da_{2}D_{6}\frac{R_{1}^{2}-1-2InR_{1}R_{1}^{2}}{4}-Da_{2}D_{7}\frac{I_{1}(t_{2})-I_{1}(t_{2}R_{1})R_{1}}{t_{2}}+Da_{2}D_{8}\frac{K_{1}(t_{2})-K_{1}(t_{2}R_{1})R_{1}}{t_{2}}\\ +\frac{Da_{2}^{2}(1-R_{1}^{2})}{t_{2}^{2}}+\frac{2t_{2}^{2}Da_{2}}{s_{2}^{2}(s_{2}^{2}-t_{2}^{2})}\left[\frac{I_{1}(s_{2})-I_{1}(s_{2}R_{1})R_{1}}{s_{2}}C_{3}-\frac{K_{1}(s_{2})-K_{1}(s_{2}R_{1})R_{1}}{s_{2}}C_{4}\right] \end{array}\right\}$
$Q_{4}=\frac{2P^{2}}{k_{2}}\left\{\begin{array}{l} C_{3}D_{7}\frac{I_{1}(s_{2})I_{0}(t_{2})s_{2}-I_{0}(s_{2})I_{1}(t_{2})t_{2}-I_{1}(s_{2}R_{1})I_{0}(t_{2}R_{1})s_{2}R_{1}+I_{0}(s_{2}R_{1})I_{1}(t_{2}R_{1})t_{2}R_{1}}{s_{2}^{2}-t_{2}^{2}}+\\ C_{3}D_{8}\frac{I_{1}(s_{2})K_{0}(t_{2})s_{2}+I_{0}(s_{2})K_{1}(t_{2})t_{2}-I_{1}(s_{2}R_{1})K_{0}(t_{2}R_{1})s_{2}R_{1}-I_{0}(s_{2}R_{1})K_{1}(t_{2}R_{1})t_{2}R_{1}}{s_{2}^{2}-t_{2}^{2}}-\\ \frac{2Da_{2}C_{3}}{t_{2}^{2}}\times \frac{I_{1}(s_{2})-I_{1}(s_{2}R_{1})R_{1}}{s_{2}}+\frac{2Da_{2}C_{4}}{t_{2}^{2}}\times \frac{K_{1}(s_{2})-K_{1}(s_{2}R_{1})R_{1}}{s_{2}}+Da_{2}D_{8}\frac{K_{1}(t_{2})-K_{1}(t_{2}R_{1})R_{1}}{t_{2}}+\\ C_{4}D_{7}\frac{I_{1}(t_{2})K_{0}(s_{2})t_{2}+I_{0}(t_{2})K_{1}(s_{2})s_{2}-I_{1}(t_{2}R_{1})K_{0}(s_{2}R_{1})t_{2}R_{1}-I_{0}(t_{2}R_{1})K_{1}(s_{2}R_{1})s_{2}R_{1}}{t_{2}^{2}-s_{2}^{2}}+\\ C_{4}D_{8}\frac{K_{1}(t_{2})K_{0}(s_{2})t_{2}-K_{0}(t_{2})K_{1}(s_{2})s_{2}-K_{1}(t_{2}R_{1})K_{0}(s_{2}R_{1})t_{2}R_{1}+K_{0}(t_{2}R_{1})K_{1}(s_{2}R_{1})s_{2}R_{1}}{t_{2}^{2}-s_{2}^{2}}-\\ \frac{2C_{3}^{2}}{s_{2}^{2}-t_{2}^{2}}\left[\frac{I_{0}^{2}(s_{2})-I_{1}^{2}(s_{2})}{2}-\frac{I_{0}^{2}(s_{2}R_{1})-I_{1}^{2}(s_{2}R_{1})}{2}R_{1}^{2}\right]-\frac{2C_{4}^{2}}{s_{2}^{2}-t_{2}^{2}}\left[\begin{array}{l} \frac{K_{0}^{2}(s_{2})-K_{1}^{2}(s_{2})}{2}-\\ \frac{K_{0}^{2}(s_{2}R_{1})-K_{1}^{2}(s_{2}R_{1})}{2}R_{1}^{2} \end{array}\right]-\\ \frac{4C_{3}C_{4}}{s_{2}^{2}-t_{2}^{2}}\left[\frac{I_{0}(s_{2})K_{0}(s_{2})+I_{1}(s_{2})K_{1}(s_{2})}{2}-\frac{I_{0}(s_{2}R_{1})K_{0}(s_{2}R_{1})+I_{1}(s_{2}R_{1})K_{1}(s_{2}R_{1})}{2}R_{1}^{2}\right]-\\ Da_{2}D_{7}\frac{I_{1}(t_{2})-I_{1}(t_{2}R_{1})R_{1}}{t_{2}}+\frac{2Da_{2}}{s_{2}^{2}-t_{2}^{2}}\left[\frac{I_{1}(s_{2})-I_{1}(s_{2}R_{1})R_{1}}{s_{2}}C_{3}-\frac{K_{1}(s_{2})-K_{1}(s_{2}R_{1})R_{1}}{s_{2}}C_{4}\right]+\\ \frac{Da_{2}^{2}(1-R_{1}^{2})}{t_{2}^{2}} \end{array}\right\}$
